# The Role of Chronic Inflammation in Obesity-Associated Cancers

**DOI:** 10.1155/2013/697521

**Published:** 2013-05-30

**Authors:** Maria E. Ramos-Nino

**Affiliations:** Department of Pathology and Department of Medical Laboratory Sciences, University of Vermont, Burlington, VT, USA

## Abstract

There is a strong relationship between metabolism and immunity, which can become deleterious under conditions of metabolic stress. Obesity, considered a chronic inflammatory disease, is one example of this link. Chronic inflammation is increasingly being recognized as an etiology in several cancers, particularly those of epithelial origin, and therefore a potential link between obesity and cancer. In this review, the connection between the different factors that can lead to the chronic inflammatory state in the obese individual, as well as their effect in tumorigenesis, is addressed. Furthermore, the association between obesity, inflammation, and esophageal, liver, colon, postmenopausal breast, and endometrial cancers is discussed.

## 1. Introduction

Cancer development is complex and involves different phases commonly referred to as initiation, promotion, and progression [1]. It is believed that during the initiation phase, genetic mutations accumulate that lead to irreversible cellular changes. Some of the most significant changes during this phase include activation of protooncogenes (e.g., *ras, bcl2, myc, abl*) and inactivation of tumor suppressor genes (e.g., *p53, Rb*) [1]. These genome-level events give a selective growth or survival advantage to the cell, which confer on cancer cells their intrinsic properties, including self-sufficient proliferation, insensitivity to antiproliferative signals, evasion of apoptosis, limitless replicative potential, sustained angiogenesis, invasion, and metastasis [2]. Tumor development is promoted by the clonal expansion of these changed cells, and it is followed by the progression phase, which involves tumor growth and metastasis [3]. 

Determining what causes a particular cancer is a complex task. Many things are known to increase the risk of cancer, including environmental pollutants [4–6], certain infections [7, 8], certain metabolic disorders [9, 10], and so forth. For example, skin cancer has been linked to radiation therapy; viral infections such as the human papilloma virus; exposure to UV radiation; aging; skin color; diet; smoking (reviewed in [11]).

Cancer cell initiation, promotion, and progression are also intimately linked to their microenvironment. The environment of the cells can directly affect their genetic make-up or, combined with genetic predisposition, help in the cancer development. The tumor infiltrate, composed of angiogenic vascular cells, lymphatic endothelial cells, cancer-associated fibroblastic cells, and immune cells, have been shown to contribute actively to tumorigenesis [12]. 

The contribution of immune cells in tumorigenesis was first addressed by Virchow in the middle of the 19th century [13]. His conclusions were based on the fact that tumors developed in the setting of chronic inflammation and that inflammatory cells were present in tumor biopsy specimens [14]. Today, chronic inflammation is increasingly being recognized as an etiology in several cancers [13, 15] (see Table 1), and most of the resulting tumors are of epithelial origin (carcinomas) [1]. Recent epidemiological data indicate that over 25% of all cancers are related to chronic inflammation [16] and it is estimated that 15% of cancer deaths are inflammation associated [17]. Some of the evidence to support this belief is the fact that some inflammatory diseases have been associated with increased risk of cancer development. For example, there is an estimate that about 15% of all malignancies worldwide are due to infections [17], and one of the mechanisms by which infectious agents may induce carcinogenesis is the production of chronic inflammation [17]. Moreover, chronic overexpression of inflammatory mediators in the cell microenvironment can lead to increased tumor initiation, promotion, and progression [18, 19]. For example, cyclooxygenase enzymes are required for the conversion of arachidonic acid to prostaglandins. COX-2 mediates the inflammatory effects of COX activity and is induced by a wide spectrum of growth factors and proinflammatory cytokines. COX-2 is overexpressed in numerous premalignant and malignant lesions, including colorectal cancer [20]. It has also been shown that inhibition of inflammatory mediators with anti-inflammatory drugs decreases cancer incidence and progression in patients with cancer [21–25].

The origin of the inflammatory tumor microenvironment is not currently clear. Two pathways have been postulated: (a) an intrinsic pathway where the source is the genetic alterations within the cancer cells and (b) an extrinsic pathway where the source could be a chronic infection, an autoimmune disease, chronic exposure to an irritant [37], or any other chronic comorbidity associated with an inflammatory process. 

Metabolism and immunity are linked in many ways. They share many bioactive molecules that have both metabolic and immune functions, like bioactive lipids, cytokines, and others. The link between metabolism and immunity, which during homeostasis is beneficial to an individual's health, can become deleterious under conditions of metabolic stress, as exemplified by the obesity-linked inflammatory diseases like diabetes, atherosclerosis [38], and cancer. 

Obesity, an abnormal or excessive fat accumulation in adipose tissues, is considered a chronic inflammatory disease [38]. The prevalence of obesity has increased dramatically over the past 30 years due to genetic, metabolic, behavioral, and environmental factors [39]. Approximately 35% of adults and 20% of children in the US are currently obese [40]. A great majority of obese individuals meet the criteria for the metabolic syndrome: (a) increased waist circumference, (b) insulin resistance, (c) hyperglycemia, (d) hypertension, and (e) hypertriglyceridemia [41]. Obesity in turn increases risk for a number of chronic diseases including type 2 diabetes, cardiovascular disease, fatty liver disease, and some forms of cancer [39]. An estimated 15%–30% of cancer deaths in the US population are attributed to excess weight [40]. Evidence has accumulated that links obesity to endometrial cancer, postmenopausal breast cancer, colon cancer, renal cell carcinoma of the kidney, liver, gallbladder, esophageal, and pancreatic cancer, with some evidence for cervical, ovarian, prostate (prognosis), and stomach cancer [40]. Recent evidence has strengthened the proposed relationship between obesity-related insulin resistance and/or diabetes mellitus and cancer. Although the precise mechanisms and pathways are uncertain, hyperinsulinemia and possibly sustained hyperglycemia are important regulators of the development of cancer [10], but there is more to this association than meets the eye. The mechanisms by which inflammation is triggered in obesity/metabolic syndrome and how that can modify the tumor microenvironment are questions that have no clear answers. This review focuses on the inflammatory process and its impact in the tumor microenvironment as potential mechanisms underlying the association between obesity/metabolic syndrome and cancer development.

## 2. Chronic Inflammation and Obesity

Acute inflammation, a physiological process generated by the body in response to injury, infection, or irritation, is vital to healing; however, when this process becomes chronic it may contribute to a variety of diseases, including cancer. 

In response to injury, infection, or irritation, the body initiates a network of chemical signals to heal the affected tissue. The inflammatory process involves activation and directed migration of leukocytes (neutrophils, monocytes, and eosinophils) from the vasculature to the site of injury. During this stage (a) adhesion molecules (L-, P-, and E-selectin) are activated that facilitate the rolling along the vascular endothelium; (b) integrins in leukocytes are activated and upregulated facilitating the immobilization of neutrophils on the surface of the vascular endothelium by tight adhesions; and (c) transmigration through the endothelium to sites of injury is facilitated by extracellular proteases, such as metalloproteinases (MMPs) [15]. The migration of leukocytes to the site of injury is orchestrated by a family of chemotactic cytokines, named chemokines. Neutrophils (or eosinophils) are the first to be recruited to the site of injury, followed by monocytes, which differentiate into macrophages. Once activated, macrophages are the main source of growth factors and cytokines which affect the local microenvironment. Mast cells also contribute to inflammatory mediators, such as histamine, cytokines, and proteases, as well as lipid mediators [15]. Many of the same molecular mediators are generated in both acute and chronic inflammations [1]. 

Obesity is associated with a low-grade chronic inflammation, characterized by increased circulating fatty acids, and chemoattraction of immune cells that contribute to the inflammatory condition [42]. Although the features of chronic inflammation in obese adipose tissue are clearly defined, the signals and mechanisms that trigger chronic inflammation are not well understood.

## 3. Adipose Tissue

Adipose tissue cells are embedded in a connective tissue matrix and contain a vast variety of cell types including preadipocytes, adipocytes, immune cells, and endothelial cells [43]. There are two types of adipose tissue: the brown adipose tissue (BAT) and the white adipose tissue (WAT). In humans, BAT is mainly an infancy-associated fat that specializes in generating heat. In adults, it has been located around the neck and large blood vessels of the thorax [43]. WAT, on the other hand, is the main source of energy reserves and the most common adipose tissue in adults. WAT constitutes the major source of fatty acids in the body, used as energy substrate for the generation of adenosine triphosphate (ATP) through oxidative phosphorylation [44]. WAT seems to have few key functions including the control of the metabolism through energy homeostasis, adipocyte differentiation, and insulin sensitivity [45, 46]. In healthy, nonoverweight humans, white adipose tissue composes as much as 20% of the body weight in men and 25% of the body weight in women. Adipose tissue can be classified as subcutaneous and as visceral adipose tissue. The first is not related to many of the classic obesity-related pathologies, such as heart disease, cancer, and stroke, and some evidence even suggests that it might be protective around internal organs [47]. The visceral adipose tissue, on the other hand, is more predictive of obesity-associated comorbidity and mortality [42]. The adipocyte secretome and receptors are expressed differently in different adipose tissues. 

## 4. The Adipocyte

The WAT's adipocyte or fat cell main function is lipid storage. In order to accommodate the lipids, adipocytes, which vary in size (20–200 *μ*m in diameter), are capable of changing their diameter 20-fold and their volume by several-thousand-fold [48]. More recently, adipocytes have recently been implicated in the modulation of a range of physiological responses, including lipid metabolism, glucose homeostasis, inflammation, angiogenesis, hemostasis, and blood pressure. 

### 4.1. Adipogenesis

Adipocyte precursor cells emerge from mesenchymal stem cells. The pluripotent MSCs receive extracellular signals that lead to the determination of the preadipocyte linage. Adipogenic differentiation is characterized by arrested growth of proliferating preadipocyte and the increase expression of key adipocyte markers such as fatty acid-binding proteins, lipoprotein lipase, CCAAT/enhancer binding protein alpha (C/EBP*α*), and peroxisome proliferator-activated receptor gamma (PPAR*γ*) [49]. The later phase of adipogenesis is referred to as terminal differentiation. 

It has long been proposed that new adipocytes arise solely from resident preadipocytes progenitors, but accumulating evidence points toward a contribution from outside sources, in particular the bone marrow [50, 51]. 

### 4.2. Fat Storage and Release

**Figure 1:**
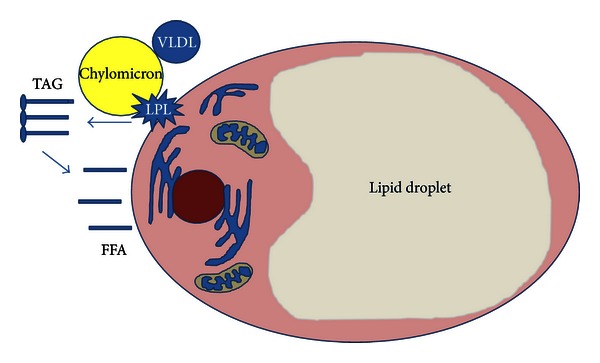
The WAT's adipocyte cell storage triglycerides (TAGs). Approximately 90% of the adipocyte is a lipid droplet. The steps for lipid storage. (a) The adipocyte releases lipoprotein lipase (LPL) to the environment to break down triglyceride molecules presented by the chylomicrons and the very low density lipoprotein (VLDL) into glycerol and free fatty acids. The free fatty acids enter the cell and are reformed into TAGs.

The adipocyte stores triacylglicerides (TAGs). Approximately 90% of the adipocyte is a lipid droplet, and the remaining 10% consist of cytoplasm, mitochondria, nucleus, and other organelles (Figure 1). The steps for lipid storage and release depend on several critical molecules. The adipocyte releases lipoprotein lipase (LPL) to the environment to break down triglyceride molecules presented by two lipid transport molecules, the chylomicrons and the very low density lipoprotein (VLDL) into glycerol and free fatty acids. The free fatty acids enter the cell and are reformed into TAGs. Lipid release from the adipocyte was believed to be triggered via hormonal activation of hormone-sensitive lipase (HSL). However, recent studies on HSL-null mice have challenged this concept [52]. Although HSL-mediated lipolysis is a significant contributor to free fatty acid liberation from the adipocyte, other TAG lipases have been identified including desnutrin/ATGL [52]. Lipolysis is under tight hormonal regulation, with insulin as an inhibitor and catecholamines, as well as potentially other factors, as stimulants of lipolysis in the adipocyte [52].

**Figure 2:**
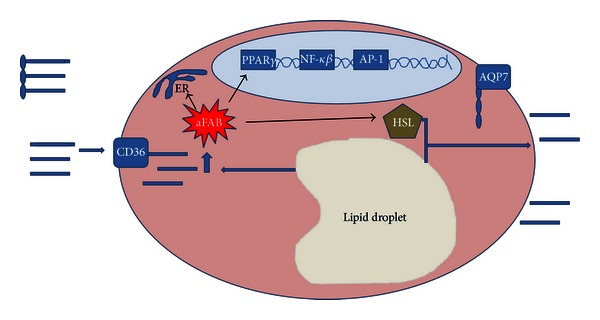
Important molecules during fat release by the WAT's adipocyte. Lipid release from the adipocyte is in part triggered via (a) hormonal activation of hormone-sensitive lipase (HSL); (b) the adipose fatty acid binding protein (aFABP), a carrier protein for free fatty acids, eicosanoids, and retinoids, thought to facilitate the transfer of fatty acids between extra- and intracellular membranes, and lipophilic molecules from outer cell membrane to intracellular receptors such as PPAR; (c) aquaporin 7 which exports the glycerol molecule released from TAGs; and (d) CD36 which facilitates free fatty acid transport through the plasma membrane.

Other molecules of importance during fat uptake and release by the adipocyte are the lipid droplet-associated protein Perilipin, which restricts access of TAG lipases during the unstimulated state [53]; the adipose fatty acid binding protein (aFABP), a carrier protein for free fatty acids, eicosanoids, and retinoids, thought to facilitate the transfer of fatty acids between extra- and intracellular membranes [54, 55] and lipophilic molecules from outer cell membrane to intracellular receptors such as PPAR [56]; aquaporin 7 which exports the glycerol molecule released from TAGs [57]; and CD36 which facilitates free fatty acid transport through the plasma membrane [58] (see Figure 2).

### 4.3. The WAT's Adipocyte Secretome and Receptors

**Figure 3:**
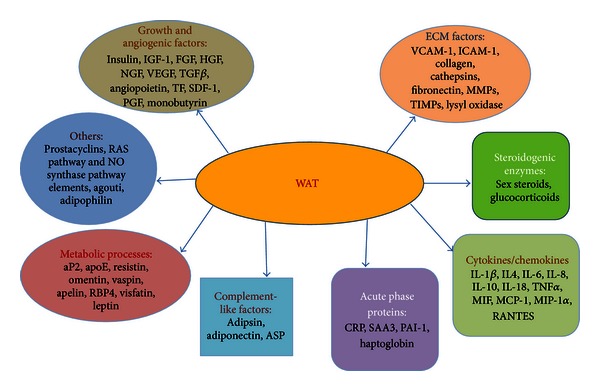
Signals emanating from the white adipose tissue. ASP: acylation-stimulating protein; aP2: activating protein 2; apoE: apolipoprotein E; RBP4: retinol binding protein 4; RAS: rennin-angiotensin system; NO synthase: nitric oxide synthase; IGF-1: insulin-like growth factor 1; FGF: fibroblast growth factor; HGF: hepatocyte growth factor; NGF: nerve growth factor; VEGF: vascular endothelial growth factor; TGF*β*: transforming growth factor beta; TF: tissue factor; SDF-1: stromal derived factor; PGF: placental growth factor; VCAM-1: vascular cell adhesion molecule 1; ICAM-1: intracellular adhesion molecule 1; MMPs: matrix metalloproteinase proteins; TIMPs: tissue inhibitor of metalloproteinase; IL: interleukin; TNF*α*: tumor necrosis factor alpha; MIF: macrophage migrating inhibitor factor; MCP-1: monocyte chemotactic protein-1; MIP-1*α*: macrophage inflammatory protein 1; RANTES: Regulated on Activation, Normal T cell Expressed and Secreted; CRP: C-reactive protein; SAA3: serum amyloid A3; PAI-1: plasminogen activator-1.

The first signaling molecule found to originate from the adipocyte was Leptin in 1994 [59–64]. Since then, a vast array of signaling molecules has been added to that list (Figure 3). Similarly, the adipocyte expresses plasma membrane receptors that include the hormone-cytokine receptor, thyroid-stimulating hormone, glucagon, IL-6, TNF*α*, gastrin/cholecystokinin-B, neuropeptide Y-Y1, atrial natriuretic peptide, epidermal growth factor, platelet-derived growth factor, fibroblast growth factor, gastrin inhibitory peptide, glucagon like peptide-1, angiotensin II, leptin (OB-R), growth hormone, prostaglandin, adenosine, lipoprotein lipase and insulin; the lipoprotein receptor, very low density lipoprotein, low-density lipoprotein, and high-density lipoprotein; the catecholamine-nervous system receptors, *β*1, *β*2, *β*3, *α*1, *α*2; and the nuclear receptors, peroxisome proliferator-activated receptor gamma (PPAR*γ*), retinoic acid receptors (RAR and RXR), estrogen, androgen, vitamin D, thyroid hormone, progesterone, and glucocorticoids (summarized in [48]).

## 5. Obesity

Obesity, a medical condition in which people's body mass index (BMI) exceeds 30 kg/m^2^, is the sixth most important risk factor contributing to the overall burden of disease worldwide [65]. Obesity is associated with a chronic state of inflammation, but the causes and mechanisms involved in obesity-induced inflammation are not fully understood. Accumulating evidence indicates that obesity-induced inflammation can be in part attributed to increased fatty acids; inflammatory cytokine production of the expanding adipose tissue; the influx of immune cells that add to the production of inflammatory mediators [42]. 

Under normal conditions, adipocytes store lipids and regulate metabolic homeostasis. Under these conditions, resident tissue macrophages present a predominant polarization of M2 type, release mainly anti-inflammatory cytokines [66, 67] like IL-10, IL-1R*α*, and the enzyme arginase, involved in the inhibition of nitric oxide synthase (iNOS) [66, 68]. Normal WAT potentially contributes to this anti-inflammatory environment through peroxisome proliferator-activated receptors (PPARs) and liver X receptor (LXR) cell signaling [69, 70]. In obesity, WAT becomes an inflammatory source and the change in the cytokine profile induces the resident macrophages to a more activated M1 type (CD11 c+) [66, 68, 71, 72]. M1 macrophages release iNOS and proinflammatory cytokines [72]. However, macrophage subsets in WAT show no strict M1 or M2 subtypes [66, 73]. Beside the resident macrophages, an increased influx of macrophages occurs in WAT during obesity, which exacerbates the inflammatory state [74, 75]. The obesity-induced inflammatory cytokines seem to be responsible for the activation of adhesion molecules in endothelial cells and the recruitment of monocytes and macrophages [76]. In addition to macrophages, other immune cells localized to adipose tissue in obesity include neutrophils, mast cells, natural killer T cells, and lymphocytes. Whether the infiltration of these other immune cells is causal to, or result of, an increased inflammatory environment seen in obesity is not known [77]. Adipocytes from visceral body depots show a more inflammatory profile than those from subcutaneous fat [78]. As the demand for fat storage increases, obesity brings about hyperplasia as well as hypertrophy of the adipose tissue [73, 79, 80]. Adipocyte hypertrophy is induced by two factors: increased fat storage in differentiated adipocytes and increased expression of proinflammatory mediators [80, 81]. As the process from lean to the obese state occurs, radical changes happen in the adipocyte microenvironment, as well as the intracellular adipocyte state, including hypoxia, endoplasmic reticulum stress, and mitochondrial stress that result in insulin resistance, changes in the adipocyte secretome, free fatty acid dysregulation, and chronic inflammation.

### 5.1. Adipocyte Microenvironment

As the adipose tissue expands, a partial break down of the extracellular matrix (ECM) is required in order to prevent the extracellular matrix from restraining the expanding adipocytes. Failure to do so could result in problems such as ectopic lipid deposition and lipotoxic effects in organs like the liver, muscle, and pancreas [82]. Key extracellular components during these changes are, among others, those associated with fibronectin in the ECM. Cathepsin S, an adipokine and cysteine protease involved in the degradation of fibronectin, is upregulated in the obese state and is involved in the differentiation of human preadipocytes [83]. Another upregulated protease, Cathepsin K, can degrade collagens and is required for the induction of lipid storage program during 3T3-L1 differentiation [84]. Some matrix metalloproteases (MMPs) and their control regulatory protein, the tissue inhibitory of matrix metalloproteinases (TIMPs), are also secreted by the adipocyte and dysregulated in the obese state, although the role of these proteases and protease- inhibitors in obesity is not clear [43].

### 5.2. Hypoxia

In order for growing fat mass to sustain its growth, ongoing neovascularization is also required. New endothelium can be made of endothelial cells already present in the adipocyte microenvironment or by the maturation of new endothelial cells from circulating endothelial progenitors [85, 86]. Strong evidence on the effect of angiogenesis on adipose tissue expansion comes from studies where the use of angiogenesis inhibitors triggered a reduction in fat mass [87–89]. The adipocyte secretes a vast array of factors that modulate angiogenesis (see Figure 3). Nevertheless, a rapidly expanding fat mass still experiences hypoxia [90–92]. During hypoxia a large amount of proangiogenic molecules are secreted by the adipocyte [93, 94] and the macrophages in its microenvironment [91, 95]. Macrophages have a very active role in angiogenesis under normoxic condition but become even more active during hypoxia. Recently it has been observed that obesity induces a phenotypic switch from the M2 phenotype involved in tissue remodeling, angiogenesis, and a type 2 inflammatory response to the M1, a phenotype involved in the killing of intracellular parasites and type 1 inflammatory response, thereby decreasing the angiogenic potential of the macrophages in adipose tissue [96]. 

The role of hypoxia in chronic inflammation in adipose tissue was first proposed by Trayhurn and Wood [97]. Recent studies have provided consistent evidence that adipose tissue hypoxia exists and that it contributes to initiation of chronic inflammation and inhibition of adiponectin expression in the white adipose tissue [90–92, 98–100]. 

### 5.3. Reactive Oxygen Species (ROS)

Oxidative stress is caused by an imbalance between increased production of reactive oxygen species (ROS) and reduced antioxidant activity, leading to oxidative damage to cells [101]. It is well known that oxidative stress is involved in the pathology of several diseases, including cancer, diabetes mellitus, hypertension, and cardiovascular diseases [101, 102]. Several studies have suggested that obesity is associated with increased oxidative stress [103–107] and inversely correlated to antioxidant capacity [108, 109]. Some of the potential mechanisms of obesity-associated oxidative stress are, among others, the adipose tissue itself. An inflammatory process, especially in visceral obesity, is observed as WAT mass increases. This inflammatory process is activated in the WAT itself, liver, and immune cells [71, 74, 110, 111]. This response determines an increase in circulating levels of proinflammatory cytokines, hormone-like molecules, and other inflammatory markers [71]. These adipokines in turn are stimulators for the production of reactive oxygen species and nitrogen species by macrophages and monocytes [112, 113]. Adipose tissue also produces angiotensin II, which stimulates nicotinamide adenine dinucleotide phosphate (NADPH oxidase) activity, one of the major sources for ROS production in adipocyte [102, 114]. Also, free fatty acids can contribute to oxidative stress, as described in the next section. Several studies have demonstrated that obesity could also deplete antioxidant sources, decreasing the activity of enzymes such as superoxide dismutase (SOD), glutathione peroxidase (GPx), and catalase (CAT) [113].

### 5.4. Free Fatty Acids

WAT is a major source of nonesterified fatty acids, also called free fatty acids (FFAs), used as energy substrate for the generation of ATP after oxidative phosphorylation. Adipocytes of obese people have reduced insulin receptors and an increase in beta-3 adrenergic receptors, which contribute to the increase in lipolysis rate [113]. Hyperlipidemia leads to increased uptake of fatty acids by skeletal muscle, liver, heart, and pancreatic *β*-cells [115]. These nonadipose tissues are less capable of storing lipids than the adipocytes which increase the accumulation of toxic fatty acid metabolites that stimulate inflammation and inhibit insulin signaling [116]. Excessive fat accumulation can also cause cellular damage, as in nonalcoholic steatohepatitis [113]. The cellular damage leads to production of cytokines such as TNF*α*, which in turn generates ROS in the tissues, increasing the lipid peroxidation rate [117]. 

Mitochondrial and peroxisomal oxidation of fatty acids is capable of producing free radicals in cells, which could result in mitochondrial alterations. The mitochondrial function is key for the proper maintenance of energy homeostasis. Even small changes in the level of mitochondrial function have a dramatic effect on production and release of adipokines in the adipocytes, particularly adiponectin [118], an adipokine involved in anti-inflammatory, antioxidant, and other processes [71]. 

Lipids can also be anti-inflammatory. Ligands of the LXR and PPAR families of nuclear hormone receptors are oxysterols and fatty acids, respectively, and activation of these transcription factors inhibits inflammatory gene expression in adipocytes and macrophages, largely through suppression of NF*κ*B [69, 70, 119, 120]. 

An example of inflammatory fatty acids is arachidonic acid (n-6 fatty acid), a precursor of immune-active mediator known as eicosanoids (lipoxins, leukotrienes, and prostaglandins) (reviewed in [121]). On the other hand, n-3 fatty acids and some of its derivatives, including resolvins (generated from n-3 fatty acids docosahexaenoic acid (DHA) and eicosapentaenoic acid (EPA)), have potent anti-inflammatory and immunoregulatory actions. In the case of resolvins, they can prevent neutrophil entry to inflammation sites and cytokine production [122]. Some studies demonstrate an increase in resolving D1 and 17-hydroxy-DHA, a marker of resolving synthesis, in adipose tissue of obese-diabetic mice fed with n-3 fatty acids [123]. Other n-3 fatty acid-derived lipid mediators like protectin D1 and other aspirin-triggered lipoxins are produced after acetylation of COX-2 by aspirin and help in the anti-inflammatory process by inhibition of neutrophils tissue infiltration and stimulation of macrophage phagocytosis of apoptotic neutrophils [124].

The role of lipids in inflammation depends on the location in the body, the composition of the microenvironment, and their coupling to target signaling pathways [38]. 

### 5.5. Endothelial Reticulum Stress (ER)

The ER, an organelle in which proteins are synthesized, folded, and matured [125], is responsive to cellular nutrient and energy status [77]. Recent studies have suggested that ER stress and the unfolded protein response (UPR), a system that mitigates ER stress, are activated under obese conditions [38, 126–129]. Three major transducers of the UPR have been identified, including PKR-like ER kinase (PERK), part of the family of eIF2*α* kinases whose activation results in expression of proapoptotic transcriptional factors CCAAT/enhancer binding protein (C/EBP), homologous protein (CHOP), and growth arrest and DNA damage-inducible protein 34 (Gadd 34) [130, 131]; inositol-required enzyme 1 (IRE1), involved in splicing of X-box binding protein-1 (XBP-1) mRNA, and translation of the spliced form, which in turn regulates the expression of ER chaperons and proteins involved in ER-associated degradation [132]; and activating transcription factor 6 (ATF6), that translocates to the Golgi Apparatus in response to ER stress where it is cleaved. The N-terminal fraction regulates ER chaperone expression [133]. These factors in turn activate three pathways: (a) suppression of protein translation, (b) induction of genes encoding ER molecular chaperons (BiP (GRP78), ORP150 (oxygen regulated protein 150), calnexin, calreticulin, and so forth) to facilitate protein folding, and (c) ER-associated degradation to reduce unfolded protein accumulation in the ER [134]. UPR failure induces apoptosis in cells [134]. Studies have demonstrated that FFAs have the potential to induce ER stress in adipocytes [135, 136] and the use of chemical chaperones, that alleviate ER stress, suppressed the inflammatory response and improved insulin resistance in adipose tissue [136]. 

ER stress and all three arms of the UPR are linked to major inflammatory and stress-signaling pathways, including the activation of JNK-AP-1 and IKB kinase nuclear factor kB (IKK- NF*κ*B) and the production of ROS which are pathways that play a central role in obesity-induced inflammation [128, 137, 138] and probably other pathways as well [77]. 

## 6. Obesity, Chronic Inflammation, and Cancer

Worldwide there are 1.1 billion overweight people with a BMI between 25 kg/m^2^ and 30 kg/m^2^ and 312 million with a BMI > 30 kg/m^2^ [65]. The American Cancer Society calculates that currently new cancer cases are in the order of 1.5 million with 0.5 million cancer deaths per year, nearly 1 in 5 due to obesity [139]. A large number of epidemiological studies link obesity/metabolic syndrome/diabetes-associated diseases to an increased risk for the development of several types of cancer, particularly gastrointestinal, glandular, and reproductive tract cancers [140, 141]. In addition, obesity can lead to poorer treatment outcomes, worsened prognosis, and mortality [142–146].

A comprehensive systematic review of the evidence by the World Cancer Research Fund (ECRF) and the American Institute for Cancer Research (AICR) concluded that obesity is an established risk factor for several cancers [141]. In a standardized meta-analysis of prospective observational studies by Renehan et al., 2008 (1966–2007: 221 datasets; 282, 137 incident cases; 20 cancer types), quantifying associations between a 5 kg/m^2^ increase in BMI and risk of incident cancer, showed that, in men, the increase was associated with (a) oesophageal adenocarcinoma (RR 1.52, *P* < 0.0001); (b) thyroid (1.33, *P* = 0.02); (c) colon (1.24, *P* < 0.0001); and (d) renal (1.24, *P* < 0.0001) cancers. In women, the association was found for (a) endometrial (1.59, *P* < 0.0001); (b) gallbladder (1.59, *P* = 0.04); (c) oesophageal adenocarcinoma (1.51, *P* < 0.0001); and (d) renal (1.34, *P* < 0.0001) cancers. Weaker positive associations (RR < 1.20) were found in men with rectal cancer and malignant melanoma and in women with postmenopausal breast, pancreatic, thyroid, and colon cancers. They also found an association for both sexes with leukemia, multiple myeloma, and non-Hodgkin lymphoma. The associations were generally similar in studies from North America, Europe, and Australia, as well as the Asia-Pacific region [147]. Studies with long-term followup of patients undergoing bariatric surgery for morbid obesity showed a reduction in cancer incidence in women associated with sustained weight loss supporting a causal association between obesity and cancer risk [148, 149].

One of the major challenges in the association of obesity and cancer has been linking the epidemiology with the biological basis. Biological mechanisms underlying the relationship between obesity and cancer are poorly understood. Of the most studied candidates for this association are the energy balance-associated factors (adipokines, growth factors, hormones, and their cell signaling pathways) [141], and other emerging candidates include obesity-associated hypoxia, genetic susceptibility, adipose stromal cells, and inflammatory processes [150]

## 7. Energy Balance-Associated Factors

Several energy balance-related factors are known to influence tumor progression and these have been implicated as contributors to the effects of obesity on cancer outcome. These factors include leptin, adiponectin, steroid hormones, insulin, insulin-like growth factor-1, and sirtuins [151, 152]. 

### 7.1. Leptin

A peptide hormone is encoded by the *ob* gene, which is produced primarily by WAT, but it can also be secreted by cells of the placenta, ovaries, mammary epithelium, brown adipose tissue, skeletal muscles, the fundal glands of the stomach, bone marrow, pituitary, and the liver [153]. Leptin signals through the leptin receptor (LEPR), encoded by the *db* gene, and different variants are produced through alternative splicing of the gene. The long form of the receptor, LEPR-B, has a cytoplasmic domain that transduces the leptin-mediated signaling [154]. Leptin is involved in hypothalamic regulation of body weight and energy balance by promoting a sensation of satiety [40]. Genetic loss-of-function mutants for leptin or its receptor in mouse models (i.e., ob/ob or db/db mice) develop systemic metabolic abnormalities that include obesity, diabetes, infertility, and immune defects [155]. In the obese state, leptin is overproduced and leptin resistance usually develops [40, 156]. Increased concentrations of leptin in the obese are associated with greater amounts of adipose tissue [157, 158] that in turn affect immune function, cytokine production, angiogenesis, carcinogenesis, and other processes [159]. Leptin has been extensively studied as a potential mediator of obesity-associated cancer [160]. Leptin signaling plays an important role in tumor cell growth and survival that may be mediated through a set of responses of LEPR-positive tumor cells [154] including cancer stem cells [161]. It is activated by insulin, glucocorticoids, tumor necrosis factor-alpha (TNF*α*), and estrogens [159] and induces cancer progression by activation of the JAK2/STAT3 [162], PI3K, and MAPK pathways [163–166] through LEPR-B. A number of studies indicate that LEPR are overexpressed in many tumor tissues and that there are leptin-responsive tumors including mammary carcinomas, pancreatic, esophageal, gastric, and colon tumors [167–170]. Leptin triggers cell proliferation, migration, and invasion in different cell types [154, 171], antiapoptotic and proangiogenic effects, alone or in synergy with vascular endothelial growth factor (VEGF) [172, 173], and is a proinflammatory agent [150], inducing T helper 1 cells and potentially contributing to the progression of autoimmune responses [174].

### 7.2. Adiponectin

A peptide hormone, secreted mostly from visceral adipose tissue, is involved in energy homeostasis, carbohydrates, and lipid metabolism [175, 176]. This hormone is present in plasma as two epimers: low molecular weight (LMW) and high molecular weight (HMW) [177, 178]. The HMW forms of adiponectin predominate in the serum of healthy individuals and are normally decreased in obesity [139, 179, 180]. Furthermore, high levels of adiponectin have been associated with low body fat [181, 182]. Adiponectin improves fatty acid catabolism [183]; increases insulin sensitivity, possibly as a result of its role in lipid peroxidation, improvement of insulin signaling, inhibition of TNF*α*, or/and inhibition of gluconeogenesis [184]; and exhibits anti-inflammatory characteristics, possibly by suppressing the migration of anti-inflammatory mediators such as monocytes and macrophages [184]. Adiponectin may exert part of its anticancer effects by (1) decreasing insulin/insulin-like growth factor-1 (IGF-1) secretion, (2) modulating mTOR signaling by activating AMP-activated protein kinase (AMPK) and peroxisome proliferator-activated receptor PPAR*γ* metabolic pathways, leading to an increase in fatty acid oxidation, glucose uptake, and a decreased rate of gluconeogenesis, thus enhancing insulin sensitivity, and (3) exerting anti-inflammatory action via the inhibition of nuclear factor kappa-light-chain-enhancer of activated B cells (NF-*κ*B) [185]. Activation of NF-*κ*B is a potential mechanism through which inflammation may stimulate cancer development [42, 152]. Several studies have suggested that higher levels of adiponectin are associated with higher levels of high density lipoproteins (HDL) and lower levels of low density lipoprotein (LDL), triglycerides (TGs), and total cholesterol [186]. Dyslipidemias, like low HDL, high LDL, and high TGs levels, are associated with some cancers including lung, non-Hodgkin lymphoma and have been suggested to be a marker for increased breast cancer risk, since they may reflect unfavorable hormonal profile with increased estrogen levels in obese women [187–190]. High serum levels of cholesterol and TGs raise the risk of prostate cancer and postmenopausal breast cancer [191, 192]. Furthermore, adiponectin plays a role in the secretion of estrogen [193] and estrogen plays a role in cancer development, as discussed later. 

In population-based studies, it has been found that adiponectin levels are inversely associated with increased risk of cancer, including endometrial, breast (postmenopausal), colon, esophageal, prostate cancers, and pancreatic cancer in men [177, 194–200]. Most studies suggest that adiponectin may have protective effects against the development of cancer, and that the association is correlated with estrogen, IGF, obesity, and insulin resistance. Further investigations are needed to clarify these associations.

### 7.3. Steroid Hormones

Steroid hormones, including adrenal steroids, androgen, progesterone, and estrogen, are associated with energy balance and obesity-associated progression of several cancers [201]. Adipose tissue can produce estrogens in men, postmenopausal or ovarian-hormone-deficient women, via aromatase-catalyzed conversion of gonadal and adrenal androgens [141, 202]. Obesity also increases the bioavailability of estradiol by reducing the production of sex hormones-binding globulin (SHBG) [139, 202], raising the risk of postmenopausal breast, endometrial, and colon cancers [202]. In support of this, data from the Endogenous Hormones and Breast Cancer Collaborative Group (EHBCCG) [203], a pooled analysis of nine prospective studies and the European Prospective Investigation into Cancer and Nutrition (EPIC) study [204], demonstrate that postmenopausal breast cancer risk is increased among women with higher concentrations of circulating sex steroids and lower levels of SHBG. Adiposity has been inversely related to testosterone concentrations in men [205] but positively related in women [203]. However, the experimental evidence in women is conflicting [150]. Similar to breast cancer, epidemiological studies have shown that higher levels of estrone and estradiol are associated with increased endometrial cancer risk in postmenopausal women [202]. Androgenic and estrogenic steroids have also been identified as key risk factors in the etiology of colorectal cancer (CRC). In population studies, women taking hormone replacement therapy have been found to have a reduced colorectal cancer risk [206, 207]. Furthermore, genetic polymorphisms in both androgen and estrogen receptors are associated with altered CRC risk [208]. 

The role of estrogen in inflammation is complex. On one hand, studies have observed suppression of inflammation with increased estrogen in several animal models of chronic inflammatory diseases. On the other hand, there is evidence of proinflammatory effects in some chronic autoimmune diseases in humans [209]. The effects of estrogens are dependent on criteria such as (1) the immune stimulus (foreign antigens or auto antigens); (2) the cell types involved during different phases of the disease; (3) the target organ with its specific microenvironment; (4) the reproductive status of a woman; (5) the concentration of estrogens; (6) the variability in expression of estrogen receptor *α* and *β* depending on the microenvironment and the cell type; and (7) intracellular metabolism of estrogens leading to important biologically active metabolites with quite different anti- and proinflammatory functions (reviewed in [209]). The concept that estrogens have anti-inflammatory, but also proinflammatory roles, depending on afore-mentioned criteria, makes it difficult to elaborate on its effect in obesity-associated cancers. It is known that estrogen represses IL-6, a proinflammatory and anti-inflammatory cytokine, through an ER-dependent mechanism, and that serum levels of IL-6 increase following menopause, in healthy women, and with age in both men and women [210, 211].

### 7.4. Insulin and Insulin-Growth Factor-1

Insulin is a peptide hormone produced by the beta cells of the pancreas and released in response to elevated blood glucose. In the obese state, blood glucose levels increase and trigger the pancreas to increase insulin production, resulting in hyperinsulinemia, hyperglycemia, and insulin resistance [212–214]. The development of insulin resistance is linked to chronic inflammation and the production of adiponectin and IGF-1 [215–217]. The insulin growth factor-1 system comprises three peptides, insulin, IGF-1, IGF-2 and each of its receptors (IR, IGF-1R, IGF-2R), as well as at least six IGF-binding proteins (IGFBPs). IGF-2 is a fetal growth factor [218] while IGF-1 stimulates fetal as well as postnatal growth [139]. IGF-1 is a peptide growth factor that shares approximately 50% sequence homology with insulin and is produced primarily by the liver following stimulation mainly by growth hormone, as well as hyperinsulinemia and hyperglycemia [40]. IGF-1 circulates bound to IGFBPs, and when free, binds to its IGF-1R, eliciting growth and survival signaling [219]. The growth promoting effects of IGF-1 include proliferation, differentiation, protein synthesis, modulation of cyclins and cyclin-dependent kinase inhibitors [220], proangiogenic action [221], and inhibition of apoptosis [139, 222]. Similar to insulin, levels of IGF-1 correspond to energy status and are often elevated in obesity [145, 223], possibly via hyperglycemia-induced suppression of IGFBPs synthesis and/or growth hormone receptor expression and IGF-1 synthesis [219, 224]. However, the relationship between BMI and circulating IGF-1 and IGFBPs is complex and nonlinear [225]. Hyperinsulinemia increases the risk for colorectal, kidney, breast, endometrial, and pancreatic cancers [226–229]. The proliferative effects of insulin are believed to be an indirect effect through increasing levels of bioavailable IGF-1 [42, 230], and the role of IGF-1 as a risk factor for cancer has been well established [231–234].

The signaling processes downstream of IGF-1R activation are similar to those of insulin and involve two major signaling pathways, the mitogenic extracellular signal-regulated kinase (ERK) and the metabolic and antiapoptotic phosphatidylinositol-3-kinase (PI3K) pathways, important to the modulation of transcription factors that control gene expression related to cancer development [42, 235]. Activation of the insulin receptor (IR) and the IGF-1R stimulate PI3K, which in turn activates Akt, a regulator of the mammalian target of rampamycin (mTOR). mTOR activation results in protein synthesis, preparation of mitosis through S6k1 and 4E-BP-1, and cell growth, all processes that support tumor growth [236]. mTOR is inhibited by two tumor suppressors, phosphatase and tensin homolog (PTEN) and tuberous sclerosis (TSC) [237], and by increased AMP-activated kinase (AMPK) under low nutrient conditions and hypoxia [238]. PTEN is one of the most commonly mutated tumor suppressor genes in human cancer. The loss of this tumor suppressor results in an increased signaling of IGF-2 through IGF-1R and IR-A [239]. IGF-1 also mediates its effect through the MEK-ERK pathway. It is conceivable that in some cell types it might be necessary to activate multiple signaling pathways at once to avoid apoptosis, and that the signals activated depend on the specific cell type [222]. Estrogen is another factor that can activate the MEK-ERK pathway. Estrogen was demonstrated to induce the expression of IGF-1R as well as the insulin receptor substrates IRS-1 and IRS-2 in breast tumor cells. This leads to the activation of MAPK after IGF-1 stimulation [240].

### 7.5. Sirtuins

Lysine acetylation/deacetylation has been recognized as an important posttranslational modification, regulating numerous cellular processes. Sirtuins, NAD-dependent lysine-deacetylases, have recently been associated with the regulation of lifespan in lower organisms and their capacity to interfere with cell growth, proliferation, and survival in response to stress [241]. Their requirement for NAD suggests that these enzymes may represent an important molecular link between metabolism and several human disorders such as diabetes and cancer [241]. Sirtuins have been associated with regulation of aging, endocrine signaling, transcription, and metabolic changes associated with obesity [152]. In mammals, sirtuin 1 (SIRT1) promotes long-term survival of cells [242]. SIRT1 is a nicotinamide adenine dinucleotide-dependent deacetylase that acts on Ku70, which in turn sequesters the proapoptotic factor Bax from the mitochondria, thus inhibiting stress-induced apoptosis [242]. Sirtuins have been shown to regulate several obesity-associated metabolic changes including regulation of adiponectin secretion [243, 244], insulin secretion and sensitivity, plasma glucose levels [245, 246], regulation of oxygen consumption, and mitochondrial capacity [247, 248]. Conflicting results exist as whether SIRT1 is a tumor suppressor gene or an oncogene [249]. SIRT1 is upregulated in several tumor types and can inhibit apoptosis and downregulate the expression of tumor suppressor genes to impact epithelial cancer cells [250]. Preclinical studies suggest that activation of SIRT1 could be a cancer prevention strategy [251]. In general, sirtuins play key roles in tumourigenesis, as some have tumor-suppressor functions and others influence tumors through their control of the metabolic state of the cell [252]. Cancers associated with Sirtuins are, among others, SIRT1 with acute myeloid leukemia, colon, bladder, prostate, ovarian and glioma cancers; SIRT2 with glioma; SIRT3 and SIRT4 with breast cancer; SIRT 5 with pancreatic and breast cancers; SIRT6 with colon and breast cancers, and SIRT7 with breast cancer [252].

Recently, the identification of several transcription factors, known to play a role in the immune system, as sirtuin substrates, has suggested that this family of enzymes may also play an important role in the regulation of inflammation, a pathological situation with clear links to metabolism. SIRT1 deacetylation of p65 lysine 310 can inhibit the recruitment of the bromodomain-containing coactivator Brd4. Lack of Brd4 recruitment is thought to impair the binding of CDK9 and the recruitment and phosphorylation of RNA polymerase II (PolII), leading to reduced transcription of several proinflammatory mediators such as interleukin IL-1*β*, IL-2, IL-6, TNF*α*, and MMP9 [241]. 

## 8. Obesity-Associated Hypoxia

Recent studies support a hypoxia response in the adipose tissue in obese animals [91]. Adipose tissue hypoxia (ATH) may provide cellular mechanisms for the development of insulin resistance, chronic inflammation, macrophage infiltration, adiponectin reduction, leptin elevation, adipocytes death, ER stress, and mitochondrial dysfunction in white adipose tissue in obesity [100, 150, 253]. Therefore, ATH might contribute to cancer risk in the obese population. Hypoxia-inducible factor 1 alpha (HIF-1*α*), the most important transcription factor regulated by hypoxia, leads to an elevation of vascularization in tumors [139]. In normoxia, levels are regulated by ubiquitination and subsequent degradation in the proteosome. H1F-1*α* upregulation results from decreased ubiquitination induced by EGF, insulin, and IGFs through their PI3K/Akt and MAPK pathways [100]. H1F-1*α* regulates the transcription of genes that are involved in different aspects of cancer biology, including angiogenesis, cell survival, glucose metabolism, and invasion [254], and has been associated with increased patient mortality [254], poor prognosis, and increased metastasis [255]. Furthermore, HIF-1*α* inhibition might improve sensitivity of tumors to radiation [256]. In 2005, HIF-1*α* was shown to be increased in adipose tissue of obese patient and its expression was reduced after surgery-induced weight loss [257]. The increase in HIF-1*α* expression was confirmed in adipose tissue and adipocytes [91, 99, 258].

HIF-1*α* pathways interact with the NF-*κ*B pathway, linking hypoxia to inflammation [256], and activation of NF-*κ*B complex is a potential mechanism through which inflammation may stimulate cancer development [42, 152].

## 9. Genetic Susceptibility

Are genetic factors that predispose to obesity related to the same factors that predispose to certain tumors? Recent studies have made progress mapping obesity-linked genes [259] with those of cancer [260–262]. A few potential overlaps have been found for breast cancer on chromosomes 11p and 16q and for colorectal cancer on 18q [150]. A recent Scottish case-control study addressing the latter found no association [263], but more studies are required to prove or disprove this hypothesis.

## 10. Adipose Stromal Cells

As tumors develop, they require the development of new vasculature that supplies them with nutrients and oxygen. Mesenchymal stromal cells might be a potential source for the formation of this neovasculature. Bone marrow has been thought to be the main source of circulating progenitor cells, but recent evidence points towards the belief that WAT could be another source [264]. Adipose tissue contains a population of tumor-tropic mesenchymal progenitors, termed adipose stromal cells (ASCs), which engraft in neighboring tumors to form supportive tumor stroma [265]. Abdominal visceral adipose tissue, particularly, may contain a uniquely tumor-promoting population of ASC [265]. When transplanted into mice, adipose stromal cells (ASCs) can serve as vascular adipocyte progenitors that promote tumor growth, perhaps helping explain the obesity-cancer link [266]. ASCs are expanded in obesity and they migrate from endogenous WAT to tumors in several mouse models of cancer [266]. Evidence is starting to accumulate that links ASCs recruited from endogenous adipose tissue by obesity-associated hypoxia and inflammation with tumor growth and development [150] to contribute to tumor growth and development.

## 11. Obesity-Related Inflammation

The links between obesity and inflammation and between chronic inflammation and cancer suggest that inflammation might be important in the obesity-cancer link [146]. In the obese state, adipose tissue is in a chronic state of inflammation. This chronic inflammation is implicated in the emergence of insulin resistance, dyslipidemia, and type 2 diabetes (T2D), as well as comorbidities, including cardiovascular disease and cancer [146, 267, 268]. This inflammation is characterized by increased serum concentrations of C-reactive protein (CRP) [217], interleukin 6 (IL-6), IL-8, monocyte chemotactic protein-1 (MCP-1), and tumor necrosis factor alpha (TNF*α*) in patients and different animal models of obesity [216, 269–271]. Interestingly, part of the adult obese population remains relatively healthy despite obesity [272–274]. The protective mechanism is attributable in part to a reduced inflammatory signaling and profile [274–276]. Brd2 hypomorph mouse studies show that blocking inflammatory signal transduction protects extremely obese animals from insulin resistance, T2D, as well as from cancer [275]. The molecular mechanisms by which obesity-induced chronic inflammation might influence tumorigenesis include increased production of proinflammatory mediators, such as cytokines, chemokines, and reactive oxygen intermediates; increased expression of oncogenes, COX-2 (cyclo-oxygenase-2), 5-LOX (5-lipoxygenase), and MMPs (matrix metalloproteinases); and proinflammatory transcription factors such as NF-*κ*B, STAT3 (signal transducer and activator of transcription 3), AP-1 (activator protein 1), and HIF-1*α* that mediate tumor cell proliferation, transformation, metastasis, survival, invasion, angiogenesis, chemo resistance, and radio resistance [277].

### 11.1. CRP

CRP is an acute-phase protein secreted mainly by the liver and an unspecific marker for inflammation, infection, and tissue injury. Given that adipose tissue secretes proinflammatory mediators, it is not surprising that CRP levels correlate with the amount of adipose tissue. It has been observed that 35% of obese men and 60% of obese women with a BMI > 30 kg/m^2^ have increased levels of CRP [278]. Moreover, expression of CRP was found in human and animal adipose tissue, showing a twofold increase in obese animals, compared to lean controls [279]. CRP was reported to predict the development of diabetes in both obese men and women [280, 281]. CRP is also associated with an increased risk to develop colorectal, cervical, and ovarian cancer [282], which are cancers that have been associated with obesity. A recent study on obesity and survival after colon cancer (388 colon cancer patients) found that patients who had the highest amounts of serum CRP were significantly more likely to die of colon cancer (*P* ≤ 0.001). The CRP levels were inversely associated with survival in American Joint Committee on Cancer stage II patients (*P* = 0.038), suggesting that CRP could be used to support treatment decisions in this subgroup. They concluded that it is obesity-related inflammation, rather than obesity itself, that is, linked with poorer survival after a colon cancer diagnosis [283]. All together, these data suggest a potential link between obesity, inflammation and cancer. 

### 11.2. IL-6

IL-6 is a cytokine produced by many tissues in the body, but adipose tissue contributes up to 35% of the circulating levels of IL-6 [284]. Levels of IL-6 correlate with weight, BMI, waist/hip circumference, waist/hip ratio, and CRP concentration [285] implicating this cytokine in obesity-associated inflammation. IL-6 signaling starts with the IL-6 receptor and subsequent phosphorylation of tumor-promoting transcription factor STAT3, proteins that directly bind to target genes, affecting translation. There is evidence implicating IL-6 in cancer tumorigenesis through the STAT3 pathway [286–288]. IL-6 has been found elevated in several cancers and cancer cell lines including colon, breast, gastrointestinal tract, lymph nodes, skin, lung, ovary, pancreas, prostate, and kidney [289–298], and it is known to promote angiogenesis [173]. One of the most compelling effects of obesity on cancer risk has been on hepatocellular carcinoma or HCC. Park et al. [299] found that obesity enhanced the development of HCC by stimulating the production of tumor-promoting cytokines IL-6 and TNF that also cause chronic inflammation. Production of these signaling molecules, which are elevated in obese mice and in humans, causes inflammation of the liver and activation of STAT3. This protein in turn activates the formation and growth of liver cancer. STAT3 activation in hepatocytes is essential for DEN-induced HCC development [300] and for obesity-stimulated tumor growth [299]. Ablation of IL-6 or TNFR1 blocked obesity-promoted hepatocarcinogenesis [301]. Recently IL-6 has been shown to contribute to systemic insulin resistance [302]. Several cytokines have strong influence on the regulation of insulin resistance in the context of hepatic inflammation. A recent study has shown that IL-6 can inhibit insulin signaling in hepatocytes [303], and insulin resistance is a potential link between obesity and cancer development.

### 11.3. TNF*α*

Tumor necrosis factor alpha is a cytokine involved in systemic inflammation and is a member of a group of cytokines that stimulate the acute phase reaction. The most abundant cellular sources of TNF*α* are macrophage and monocyte [304], but it is produced also by a broad variety of cell types, including lymphoid cells, mast cells, endothelial cells, cardiac myocytes, fibroblast, neurons, and adipose tissue [305, 306]. Obesity leads to infiltration of adipose tissue by macrophages and increased levels in proinflammatory cytokines. The first indication for increased cytokine release in obesity was provided by the identification of increased expression of TNF*α* in the adipose tissue of obese mice in the early 1990s [307]. TNF*α* is expressed in and secreted by adipose tissue, its levels correlating with the degree of adiposity [307]. In response to inflammatory stimulation, macrophage or monocyte secretes TNF*α* that can induce apoptotic or necrotic cell death of certain tumor cell lines [306]. In addition, TNF*α* is also capable of inducing cell proliferation and differentiation in many types of cells under certain circumstances [308, 309]. The increased expression of TNF*α* in adipose tissue was considered to be responsible for the development of obesity or diabetes due to the induction of insulin resistance [310] through downregulation of insulin receptors and glucose transporters [42]. TNF*α* was found to phosphorylate IRS-1 and IRS-2 and therefore interfere in the signaling of the tyrosine kinase of the IR which might also contribute to insulin resistance [139]. To further support this view, studies using mice lacking TNF*α* function showed protection from obesity-induced insulin resistance [311]. All of previous functional characteristics of TNF*α* are executed through specific members of the TNF receptor (TNFR) superfamily, mainly TNFR1, the primary receptor for soluble TNF*α*, and TNFR2, the predominant receptor for membrane-associated TNF*α*. These receptors trigger several intracellular signaling pathways, most importantly, the IkB kinase (IKK) and mitogen-activated protein kinase (MAPK) cascades, which govern gene expression through NF*κ*B and AP-1 transcription factors, respectively (reviewed in [306]). These signaling pathways, in turn, regulate cell survival, proliferation, or death. Complicated roles for TNF*α* in cancer have emerged. On the one hand, its anticancer property is mainly through inducing cancer cell death, a process that could be used for cancer therapy. On the other hand, TNF stimulates proliferation, survival, migration, and angiogenesis in most cancer cells that are resistant to TNF-induced cytotoxicity, resulting in tumor promotion. Thus, TNF is a double-edged sword that could be either pro- or antitumorigenic [312]. 

## 12. Obesity, Inflammation, and Esophageal Cancer

A marked change has been observed in the last decade in esophageal epidemiology. Whereas the incidence rates of esophageal adenocarcinoma have risen in recent decades, they remained stable for esophageal squamous cell carcinoma [313]. This rise in incidence has partly been attributed to the rise in the prevalence of obesity. Some evidence from cohorts and meta-analysis has recently confirmed the association between obesity and risk of esophageal adenocarcinoma [147, 314, 315]. Frequent gastroesophageal reflux (GER), central obesity, *H. pylori* eradication, and male gender have been identified as risk factors for Barrett's esophagus (BE), consistently found to be a strong risk factor for esophageal adenocarcinoma (EA) [316]. The best available evidence from population-based analysis suggests that the prevalence of Barrett's is 1.6% [317]. In addition, nearly half of the patients with Barrett's are asymptomatic [317]. The precise incidence of progression from Barrett's to esophageal adenocarcinoma is not known. The present hypothesis is that obesity promotes reflux, causing chronic inflammation and BE, predisposing to adenocarcinoma [318]. The degree of dysplasia is currently used as a marker for risk of progression to cancer. Intensive acid-suppression and COX-2 inhibition are potential strategies to reduce the risk of progression [317]. 

## 13. Obesity, Inflammation, and Liver Cancer

Obesity often causes a number of comorbidities, including T2D, nonalcoholic fatty liver disease (NAFLD), and the more severe non-alcoholic steatohepatitis (NASH). Recently, obesity was recognized as a major risk factor for several common types of cancer, of which liver cancer shows a large increase in risk [65]. Several epidemiological and clinical studies have confirmed the importance of obesity as an independent risk factor for hepatocellular carcinoma (HCC), the most common form of liver cancer [319, 320]. Liver inflammation has been shown to be associated with obesity-induced NAFLD, NASH, fibrosis, and cirrhosis, resulting in elevated production of various cytokines and adipokines, which have been implicated in hepatocarcinogenesis [301]. 

## 14. Obesity, Inflammation, and Colon Cancer

Obesity has been associated with higher risk of colorectal cancer. The association between BMI and risk for colon cancer is positive in men, RR = 1.24, but somehow weaker in women, RR = 1.09 [230]. The difference could be expected, since abdominal obesity, more common in men, has been shown to be more strongly associated with metabolic abnormalities than gluteofemoral obesity [321]. This hypothesis has been supported by epidemiological evidence that associates increased waist circumference or increased waist-hip ratio with colon cancer risk in men and women, whereas body weight and BMI are associated with colon cancer risk in men but not in women [322]. These epidemiological data also support insulin resistance and subsequent hyperinsulinemia as risk factors for colon cancer [323]. Increased levels of bioavailable insulin-like growth factor- (IGF-) 1, which is known to have cancer promoting effects, are related to hyperinsulinemia [324–326]. 

In experimental studies, colon cancer has also been associated with several adipokines. For example, leptin, a proinflammatory agent, directly associated with the amount of adipose tissue, is related to insulin resistance and progression of colon cancer in experimental studies [324, 327–329]. This observation is supported by population-based studies [329, 330] that demonstrated significant associations of leptin with colon cancer risk. By contrast, adiponectin, an anti-inflammatory agent, amount is decreased in obesity and is inversely associated with the development of insulin resistance [331]. There are controversial data relating low plasma adiponectin levels with higher risk of colorectal cancer in men [332, 333]. Moreover, McMillan et al. [334] reported that preoperative CRP above 10 mg/L was significantly associated with overall mortality (HR = 2.63; 95% CI, 1.42–4.88) and disease-specific mortality (HR = 3.47; 95% CI, 1.59–7.60). Several independent studies have demonstrated that CRP is an independent predictor of colorectal cancer survival [335–340]. The Glasgow Prognostic Score (GPS) is a combined score of elevated CRP (>10 mg/L) and low albumin (<35 g/L) and has been demonstrated as a predictive test for poor outcomes in a variety of cancers [341–343]. Increased GPS was significantly associated with reduced survival time of colorectal cancer patients [337, 344, 345]. Furthermore, in a recent study, regular aspirin (a nonsteroidal anti-inflammatory drug) use after diagnosis independently reduced overall mortality (HR = 0.79; 95% CI, 0.65–0.97) and colorectal cancer-specific mortality (HR = 0.71; 95% CI, 0.53–0.95) [346]. 

## 15. Obesity, Inflammation, and Breast Cancer

There is evidence that the association between obesity and breast cancer risk is dependent on the menopausal status, with stronger evidence for postmenopausal women [347]. The association of BMI with postmenopausal breast cancer risk is particularly linked to elevated blood levels of estradiol [348]. Elevated blood concentrations of androgens are also associated with increased risk of breast cancer in both pre- and postmenopausal women, and thus androgens may be potential candidates linking obesity and breast cancer [347]. In contrast to men, testosterone concentrations are positively related with obesity in women [348]. In premenopausal women, the main site of synthesis of estrogen is the ovary. In postmenopausal women, adipose tissue is the main source of the circulating estrogens. Adipose tissue produces the enzymes aromatase. Therefore, in obese women, there is an increased conversion of the androgens androstenedione and testosterone into the estrogens oestrone and oestradiol, respectively, by aromatase. 

Another potential link is that obesity, being associated with metabolic syndrome, results in increased circulating levels of insulin and insulin-like growth factor (IGF), which are associated to carcinogenesis. Several studies have shown the association between hyperinsulinemia, measured as high circulating levels of serum C-peptide, with elevated risk of postmenopausal breast cancer [349–352]. In addition, insulin resistance is an adverse prognostic factor for breast cancer [353]. 

Adipokines might also be important contributors to the association between obesity and breast cancer risk. Women with breast cancer have higher leptin plasma levels and mRNA expression in adipose tissue as compared to healthy subjects, and the blood levels of estradiol increase proportional to those of leptin [354]. Recent studies have demonstrated that leptin can modulate the activators of STAT3, AP-1, extracellular signal regulated kinase-2 (ERK2), and MAPK, all involved in the regulation of proliferation and survival mechanisms, as well as aromatase expression, estrogen synthesis, and ER activation [355, 356]. Furthermore, leptin interferes with insulin signaling, and plasma levels of leptin directly correlate with the degree of insulin resistance in patients with T2D [357], whose association with breast carcinoma has been well studied. Adiponectin, on the other hand, has opposite function to leptin. For example, adiponectin inhibits the leptin-induced production of macrophage TNF*α* [358]. Studies confirm a significant inverse correlation between serum adiponectin levels, breast cancer risk, and poor prognosis, independently from hormone receptor status [359]. Adiponectin inhibits the proliferation of several cell types and is a negative regulator of angiogenesis [360]. Furthermore, it has been shown that adiponectin activates the PPAR*γ* pathway, previously demonstrated to be important in the expression of BRCA1, a DNA damage repair protein [361]. Among the adipokines, an emerging central role in the breast cancer pathogenesis and prognosis has been recently attributed to the inflammatory mediators TNF*α* and IL-6. TNF*α* regulates IL-6 synthesis and aromatase expression in the adipose tissue [362], and it has been implicated in the development of insulin resistance, all potential promoters of breast tumorigenesis. Adipose tissue contributes up to 35% of the circulating levels of IL-6 [284]. One of the impacts of high IL-6 levels is an increase in serum CRP, an indicator of inflammation. IL-6 increases following menopause in healthy women [211]. A study by Slattery et al. found a significant association between high waist-to-hip ratio, a specific IL-6 genotype, and an increased risk of breast cancer in postmenopausal women [363]. In a recent study, adipocytes isolated from breast tumor samples overexpressed IL-6 and this was associated with tumors of larger size and with lymph-node involvement [364], implicating its potential role in invasion and metastasis. Moreover, systemic chronic inflammation mediated by IL-6 may increase the risk of breast cancer recurrence and affect its prognosis [365].

## 16. Obesity, Inflammation, and Endometrial Cancer

There is a very strong association, 2.5–3.0-fold increase in risk, between obesity and endometrial carcinoma [225, 314]. Similar to breast cancer, estrogen plays an important role in this association. Several epidemiological studies have shown a link between high levels of plasma estrone and estradiol and risk for endometrial cancer in postmenopausal women [347]. Furthermore, besides a rise in estrogens and androgens, excess weight leads to a decrease in plasma sex hormone-binding globulin [202]. In a multicenter prospective study in postmenopausal women [366], circulating estrogens and androgens were found to be positively associated with endometrial cancer risk, and an inverse association was reported for sex hormone-binding globulin. Moreover, inflammatory markers known to play an important role in the development of insulin resistance, hyperglycemia, and T2D [367] are risk factors for endometrial cancer [202]. For example, increased IL-6 concentrations have been reported in patients with endometrial carcinoma [368–371], and more recently, IL-6, CRP, and IL1Ra were reported to be significantly associated with endometrial cancer risk in a prospective study [372]. Furthermore, NF-*κ*B, transcription factor involved in the immune and inflammatory response, is aberrantly expressed in a majority of endometrial cancer tumors [373, 374]. One mechanism for the inflammation-mediated association between obesity and endometrial cancer could be the modulation of aromatase activity by cytokines within the adipose tissue [375].

## 17. Conclusions 

The links between obesity and inflammation and between chronic inflammation and cancer suggest that inflammation might be important in the obesity-cancer link. Changes in the adipose tissue during the process of going from lean to obese, including modulation of adipokine levels, hypoxia, ROS, FFA, and ER stress, might lead to a chronic state of inflammation in the obese individual. The increased risk of obesity-related cancers could be mediated in part by these changes in the adipose tissue. Some of the most important elements of this association are, among others, insulin resistance; overexpression of leptin, inflammatory cytokines, sex hormones, transcriptions factors like NF-*κ*B, AP-1, STAT3, and oxidative stress; and downregulation of the expression of anti-inflammatory factors like adiponectin and PPAR*γ*, which disrupt the balance between cell proliferation and apoptosis. Accumulating evidence indicates that chronic inflammatory states in the obese might be associated with esophageal, liver, colon, postmenopausal breast, and endometrial cancers. The association between obesity, inflammation, and other cancers like prostate, renal, gastric, pancreatic, and gallbladder has been addressed in other papers. As more data accumulates and the molecular mechanisms between some of these factors and carcinogenesis start to unravel, the prospect of anti-inflammatory cancer prevention becomes an important goal in research.

## Figures and Tables

**Table 1:** Examples of inflammation-associated cancers.

Inflammatory source	Cancer	Reference
Environmental		
Tobacco	Mouth, lung	[26–28]
Asbestos	Mesothelioma	[4]
Alcohol	Liver	[29]
Infectious agent		
Schistosoma	Colorectal, bladder	[30]
Epstein-Barr virus	Hodgkin disease	[31]
*Helicobacter pylori *	Stomach	[32]
Hepatitis B and C	Liver	[8]
Physiological/metabolic conditions		
Hashimoto thyroiditis	Papilloma thyroid cancer	[33]
Gastroesophageal reflux disease	Esophageal	[34]
Chronic prostatitis	Prostate	[35]
Obesity	Breast, liver, prostate, colon, esophageal	[36]
